# Intelligence

**Published:** 1807-09

**Authors:** 


### INTELLIGENCE.

It is well known that the exhalations from certain vegetables vitiate the air in which they are placed; and it seems probable that most of those which emit a strong odour, do this in a greater or less degree. Our Paris correspondent has favoured us with the following observations by M. Sage, Member of the Institute, on the soporific effects produced by the exhalations of saffron.

This plant, M. Sage informs us, is cultivated in great abundance in Gatinais, one of the former provinces of France, and is harvested during the Autumn.

The farmers, after carefully collecting the flowers of the saffron, carry them home, and spread them on large linen sheets in their dwelling houses. In the evening the females are employed in picking off the pistils, the odour of which, though not equally strong as after they are compleatly dried, nevertheless produces the most alarming effects on the nervous system. The disease, thus induced, is vernacularly termed the Soporific Fever; the inhabitants are never affected with it at any other period of the year except during the saffron harvest, which usually lasts one month.

The narcotic effect of this odorous emanation greatly resembles that produced by opium; it is capable of occasioning death, especially in feeble patients and children. Like the affection induced by opium, it is best remedied by the employment of vinegar, of which the following fact affords a confirmation.

Madame G———, being in the Gatinais, saw a child laid out for dead by the parents, but who was in reality only affected with the lethargic disease produced by saffron flowers; she had the good fortune to recal the infant to life by means of vinegar, gooseberry water, and the employment of friction, with flannel dipped in vinegar.

M. Sage himself once succeeded in relieving a person from a similar comatose state, who had been affected by remaining a long time in a garden abounding with poppies.

Through the medium of the same correspondent, we learn that the heat has been extremely intense during the summer, at Paris, and that tempestuous weather has occurred less frequently than usual. Among the complaints at present most prevalent in that capital are catarrhal affections; which, however, have been in general successfully combated by mild diluents, slightly acidulated, by the occasional application of vesicatories, and leeches to the pained parts of the chest, and similar means. Among young females, chlorotic affections, probably produced by the extreme heat of the season, have been also unusually frequent. In the treatment of this disease, medicine, instead of proving productive of benefit, has has appeared rather to aggravate the complaint. The exercise of dancing, in a moderate degree, while care is at the same time taken not suddenly to check perspiration; the use of tepid and acescent drinks, &c. have been found more useful in alleviating it, than the prescriptions of the apothecary.

Owing to the state of the atmosphere, Phthisis Pulmonalis appears likewise to have proved very destructive. Besides the use of lowered air, the employment of fumigations with fir shoots, on the supposition that the tuberculous affection did not extend beyond the bronchiæ, has greatly tended to relieve the patients, and retard the progress of the malady. As this disease, our correspondent adds, has of late greatly increased in frequency, it will, he hopes, attract the particular attention of the Faculty.

---

It is known that various substances diffuse, under different circumstances, a phosphoric light more or less vivid and permanent. Such are the fluate of lime, when thrown in powder on heated bodies. The Bologna phosphorus, after being exposed to light, emits it again in the dark. Some sulphurets of zinc, when strongly rubbed with hard bodies, rotten wood, certain fishes, and other animal substances, when in a state of putrescence, display also similar phenomena. The physical and mathematical class of the French National Institute has proposed as the subject of a prize, to be adjudged on the first Monday of January, 1809, the following question. "To ascertain, by experiments, the relations which subsist between the different modes of phosphorescence, and the cause to which each species is owing, excluding from examination the phenomena of this kind which are observed in living animals." The prize is a gold medal of the value of three thousand francs. The memoirs must be transmitted to the secretary of the Institute, previous to the first of October, 1808.

Some experiments have been made at Hudson's Bay with frozen quicksilver. It has been reduced to sheets as thin as paper, by beating it upon an anvil with a hammer, at the same temperature as the quicksilver. On plunging a mass of frozen quicksilver into a glass of warm water, the latter was immediately frozen, the glass was shivered into a thousand pieces, and the quicksilver became fluid again.

There are upwards of two hundred warm springs in Portugal; and it deserves to be particularly remarked, that the greater number, and the hottest of them, issue from Granite.

---

### University of Glasgow.

The Medical Lectures in the University of Glasgow will begin on Tuesday the 3d of November, at the following hours.

Dietetics, Materia Medica, and Pharmacy, by Dr. Millar, at ten o'clock in the forenoon.—Midwifery, by Mr. Towers, at eleven.—Theory and Practice of Physic, by Dr. Freer, at twelve.—Anatomy and Surgery, by Dr. Jeffray, at two o'clock afternoon. —Chemistry,

—Chemistry and Chemical Pharmacy, by Dr. Cleghorn, at seven.—Clinical Lectures on the cases of patients in the Royal Infirmary, on Thursday evening the 12th of November, at six.——Dr. Brown will commence his Lectures on Botany about the beginning of May next.

---

The Lectures at the adjoining Hospitals of St. Thomas and Guy, will commence as follows: St. Thomas's.—Anatomy and the Operations of Surgery, by Mr. Cline and Mr. Astley Cooper, Thursday, October 1st, at two o'clock; Principles and Practice of Surgery, by Mr. Astley Cooper, Monday, October 5th, at eight in the evening.

Guy's.—Practice of Medicine, by Dr. Babington and Dr. Curry, Friday, October 2d, at ten o'clock; Chemistry, by Dr. Babington, Dr. Marcet, and Mr. Allen, Saturday, October 3d, at ten o'clock; Midwifery, and Diseases peculiar to Women and Children, by Dr. Haighton, Monday, October 5th, at eight in the morning; Pathology, Therapeutics, and Materia Medica, by Dr. Curry and Dr. Cholmeby, Tuesday, October 6th, at eight in the evening; Physiology, or Laws of the Animal Economy, by Dr. Haighton, Wednesday, October 7th, at seven in the evening; Experimental Philosophy, by Mr. Allen, to begin in November; Clinical Lectures on Select Medical Cases, by Dr. Babington, Dr. Curry, and Dr. Marcet.—N. B. These several Lectures are so arranged as not to interfere in the hours of attendance; and the whole is calculated to form a complete Course of Medical and Surgical Instruction.—Terms and other particulars to be learnt from Mr. Stocker, Apothecary to Guy's Hospital, who is also employed to enter gentlemen as Pupils to such of the Lectures as are delivered at Guy's.

---

Theatre, London Hospital.—The Autumnal Course of Lectures delivered at this Hospital, will commence on the 1st of October. Theory and Practice of Physic, by Dr. Cooke; Chemistry, by Dr. Yelloly; Theory and Practice of Midwifery, by Dr. Dennison; Occasional Clinical Lectures on Surgical Cases by Sir W. Blizard and Mr. Thomas Blizard; Anatomy, Physiology, and Operations of Surgery, by Mr. Headington and Mr. Frampton; Anatomical Demonstrations and Dissections, by Mr. Frampton; Principles of Surgery, by Mr. Headington; Materia Medica, by Dr. Buxton. Further Particulars may be known by applying to Mr. Price, Apothecary, at the Hospital.

---

Theatre of Anatomy, Blenheim Street, Great Marlborough Street.—Mr. Brookes, will commence his Autumnal Course of Lectures on Anatomy, Physiology, and Surgery, on Thursday the 1st of October, at two o'clock. The Dissecting Rooms will remain open from seven in the morning till two in the afternoon; where Mr. Brookes attends.

Theatre of Anatomy, Great Windmill Street.—Mr. Wilson will begin the Winter Course of his Lectures on Anatomy, Physiology, Pathology, and Surgery, on Thursday, October the 1st. at two o'clock in the afternoon, as usual.——The Rooms for Practical Anatomy will also be opened in the morning of the 10th of October, and demonstrations will be given and continued during the Winter, at one o'clock, by Mr. Wilson or Mr. Brodie.——Plans of the Course of Lectures may be had in Great Windmill Street.

---

Mr. Accum's Lectures on Operative Chemistry and Mineralogy, exhibiting a Summary Exposition of the Processes of Experimental Chemistry, and general practical rules to be observed in the performance of Chemical Experiments; with a Summary View of Analytical Mineralogy, exemplifying the Practical Analysis of Minerals, will commence on October the 1st.——For farther particulars, a prospectus may be had at the Laboratory, No. 11, Old Compton Street, Street, Soho.

Dr. Badham's Lectures on the Practice of Physic, Chemistry, and the Materia Medica, will be commenced on Thursday morning, 7th of October, at eight o'clock, and continued at the same hour.

Dr. Bradley will commence his Autumnal Course of Lectures on the Theory and Practice of Medicine, on Monday the 5th of October.

Mr. A. Carlisle, Surgeon to the Westminster Hospital, will begin his Course of Lectures on the Art and Practice of Surgery, in all its Branches, on Tuesday, October 6, at eight o'clock, P. M. at his house in Soho Square.

Mr. Carpue will commence his Lectures on Anatomy and Surgery, on Thursday, October the 1st. The Dissecting Room is open from seven in the morning till five o'clock in the afternoon.—Particulars may be known by applying to Mr. Carpue, No. 50, Dean Street, Soho.

Dr. Clarke and Mr. Clarke will begin their Lectures on Midwifery and the Diseases of Women and Children, on Monday, October the 5th. The lectures are read at the house of Mr. Clarke, No. 10, Upper John Street, Golden Square, every Morning, at a quarter past ten o'clock till a quarter past eleven, for the convenience of students attending the Hospitals.—For particulars apply to Dr. Clarke, No. 1, New Burlington Street; and to Mr. Clarke, No. 10, Upper John Street, Golden Square.

Drs. Dennison and Squire will, on Monday, the 5th of October, begin a Course of Lectures on the Theory and Practice of Midwifery, and the Diseases of Women and Children.

Dr. Marshall intends to deliver a Course of Lectures, in October next, on Midwifery, and the Diseases of Women and Children, at his house, No. 17, Holles Street.

Mr.

Mr. MOOR, Surgeon Dentist to Her Royal Highness the Duchess of York, will commence a Course of Lectures on the Structure and Diseases of the Teeth, on the 4th of November, in which will be explained the compleat practice of the Dentist. Further particulars may be known at his house, No. 6, Palsgrave Place, Temple.

Mr. THOMAS's Lectures on the Theory and Principles of Surgery, will commence as usual early in October. Particulars may be known at his house, Leicester-place; and at the Anatomical Theatre, Windmill Street.

---

The Report of the College of Physicians has been printed in a cheap form for general circulation, and published by Mr. HARDING in St. James's Street.

Dr. JOHN REID's Treatise on Consumption has lately been translated into German by Dr. Hilmershausen, of Neustadt in the Duchy of Saxe Coburg.

Dr. BEDDOES has a work ready for publication, entitled Researches, Anatomical and Practical, on Fever, as connected with Inflammation.

The Second Part of the Medical Observer, containing an Impartial Account of Quack Medicines, copies of the specifications entered at the Patent Office; with much interesting information relative to the practice of Quacks; and Observations on the present state of the Medical Profession in Great Britain, &c. will be published on the first of October next.

Dr. HALLIDAY of Halesworth, has in the press, Observations on the Causes and Consequences of Emphyzema, which will make its appearance early in September.

Mr. TURNBULL, Member of the Royal College of Surgeons, London, is preparing a work, to be comprised in three volumes octavo, entitled, "A System of British and French Surgery, Medical and Operative, &c. &c. with plates and original delineations."

Dr. CLUTTERBUCK (author of the Treatise on Fevers) is unanimonsly appointed Physician to the General Dispensary, Aldersgate Street.

